# Preliminary Scale of Reference Values for Evaluating Reactive Strength Index-Modified in Male and Female NCAA Division I Athletes

**DOI:** 10.3390/sports6040133

**Published:** 2018-10-29

**Authors:** Christopher J. Sole, Timothy J. Suchomel, Michael H. Stone

**Affiliations:** 1 Department of Health and Human Performance, The Citadel-The Military College of South Carolina, Charleston, SC 29409, USA; 2 Department of Human Movement Sciences, Carroll University, Waukesha, WI 53186, USA; tsuchome@carrollu.edu; 3 Center of Excellence for Sport Science and Coach Education, Department of Exercise and Sport Science, East Tennessee State University, Johnson City, TN 37614, USA; stonem@etsu.edu

**Keywords:** countermovement jump, jump height, time to takeoff, force platform, athlete monitoring

## Abstract

The purpose of this analysis was to construct a preliminary scale of reference values for reactive strength index-modified (RSI_mod_). Countermovement jump data from 151 National Collegiate Athletic Association (NCAA) Division I collegiate athletes (male n = 76; female n = 75) were analyzed. Using percentiles, scales for both male and female samples were constructed. For further analysis, athletes were separated into four performance groups based on RSI_mod_ and comparisons of jump height (JH), and time to takeoff (TTT) were performed. RSI_mod_ values ranged from 0.208 to 0.704 and 0.135 to 0.553 in males and females, respectively. Males had greater RSI_mod_ (*p* < 0.001, *d* = 1.15) and JH (*p* < 0.001, *d* = 1.41) as compared to females. No statistically significant difference was observed for TTT between males and females (*p* = 0.909, *d* = 0.02). Only JH was found to be statistically different between all performance groups. For TTT no statistical differences were observed when comparing the top two and middle two groups for males and top two, bottom two, and middle two groups for females. Similarities in TTT between sexes and across performance groups suggests JH is a primary factor contributing to differences in RSI_mod_. The results of this analysis provide practitioners with additional insight as well as a scale of reference values for evaluating RSI_mod_ scores in collegiate athletes.

## 1. Introduction

When assessing athlete testing and training data for the purpose of performance monitoring, coaches and sport scientists are often faced with questions regarding the worth of the data they have collected. Questions such as, “Is that score good?” or “How does that score compare to peer and aspirant performers?” are common as athlete performance data are reviewed. Unfortunately, answers to questions such as these are not always immediately apparent. In order to find answers, or determine the worth of data, coaches and sport scientists must engage in the process of evaluation.

Although the evaluation of data can be achieved through several means, one common approach is through a norm-referenced perspective. In this form of evaluation, an individual athlete’s score is compared to other scores considered representative of a population, and are commonly presented using percentiles. Overall, norm-referenced evaluation provides coaches and practitioners with a general idea of how an individual compares to a group or population. Normative data for various fitness and performance tests have been previously published for a variety of populations [1]. Depending on the purpose of the testing, this method of evaluation can be useful, such as in talent identification, or grouping individuals based on their abilities. However, if data for a specific measure or population do not exist, this process is not possible.

With the growing interest in vertical jump testing in athlete performance monitoring [2], as well as the increased accessibility of technology such as portable force platforms [3,4,5,6,7], many new variables derived from a single vertical jump have arisen from the literature. Although the addition of new variables used to characterize jump performance is not necessarily negative, it does however present interpretation and evaluation challenges for practitioners. One such variable is reactive strength index-modified (RSI_mod_) [8]. Reactive strength index-modified can be calculated from a standing countermovement jump (CMJ) and represents the ratio of jump height (JH) to movement time, referred to as time to takeoff (TTT). Since it was first introduced [8], RSI_mod_ has been reported to be both a reliable [9] and valid [10,11] indication of the athlete’s lower-body impulsive or “explosive” ability. Furthermore, considering RSI_mod_ takes into consideration an outcome variable (i.e., JH) and a process variable (i.e., TTT), it appears to be a simple and effective means for evaluating jumping strategy and ultimately neuromuscular functional state (i.e., adaptation or fatigue) [12].

Although a variety of normative data have been published related to CMJ’s criterion variable of jump height [13,14,15,16], normative data have yet to be published on many specific CMJ variables such as RSI_mod_. If RSI_mod_ is to be effectively used in athlete performance testing and monitoring, more information is needed related to the interpretation and evaluation of this variable. The purpose of this report is twofold (1) to present a preliminary scale of reference values for RSI_mod_, and (2) to examine differences in the constituents of RSI_mod_ (JH and TTT) across the scale in an effort to provide additional context to aid in the evaluation of this variable.

## 2. Materials and Methods

### 2.1. Participants

This analysis was completed retrospectively using archived data that were collected as part of an ongoing athlete performance monitoring program [17]. Additionally, all athletes provided written informed consent at the time of data collection. The methods and scope of this analysis were reviewed and approved by the University’s institutional review board. Countermovement jump data from a total of 151 collegiate athletes (male n = 76; female n = 75) were included in this analysis. All athletes were NCAA Division I, representing a variety of sports including baseball (n = 29; height = 182.1 ± 6.2 cm, body mass 88.0 ± 9.0 kg), men’s tennis (n = 7; height = 176.9 ± 9.0 cm, body mass = 74.7 ± 9.5 kg), men’s soccer (n = 25; height = 179.5 ± 6.8 cm, body mass = 78.5 ± 9.2 kg), men’s track and field (n = 15; height = 183.1 ± 6.3 cm; body mass = 94.4 ± 29.0 kg), women’s tennis (n = 11; height = 167.6 ± 5.6 cm, body mass = 68.6 ± 12.7 kg), women’s soccer (n = 22; height = 166.1 ± 6.2 cm, body mass = 63.9 ± 8.1 kg), women’s track and field (n = 13; height = 166.5 ± 7.2 cm, body mass = 67.1 ± 20.5 kg), softball (n = 14; height = 168.5 ± 6.7 cm, body mass = 70.4 ± 10.1 kg), and women’s volleyball (n = 15; height = 175.3 ± 7.5 cm, body mass = 70.7 ± 7.7 kg). Athletes ranged in age from 18–23 years.

### 2.2. Data Collection

In order to avoid any possible influence of fatigue from training or competition, all CMJ data included in this analysis were selected from the athlete’s preseason testing sessions. All athletes were injury-free at the time of data collection. All data were collected under the same standardized testing protocol. Specifically, participants completed a general warm-up consisting of 25 jumping-jacks, one set of five repetitions of mid-thigh clean pulls with a 20 kg barbell, and three sets of five repetitions of mid-thigh clean pulls with barbell totaling 40 kg for females and 60 kg for males. Immediately following the general warm-up, participants began jump testing where they completed a specific warm-up consisting of two submaximal CMJs performed at 50% and 75% of their perceived maximum effort. Following the specific warm-up, participants performed two maximal CMJs with approximately 60 seconds of rest between each jump. Briefly, each participant stood motionless on the force platform and then received a countdown of “3, 2, 1, jump!” After the jump command, participants performed a rapid countermovement to a self-selected depth before propelling themselves upward with the intent of jumping as high as possible. To control for arm swing, all jumps were performed while holding a near-weightless (≤0.5 kg) plastic bar across the shoulders, approximately between the seventh cervical and third thoracic vertebra [9,18]. All jumps were performed on a force platform (0.91 m × 0.91 m, RoughDeck HP, Rice Lake Weighing Systems, Rice Lake, WI, USA) sampling at 1000 Hz.

### 2.3. Data Analysis

Following data collection, voltage data obtained from the force platform were converted to the vertical component of the ground reaction force using laboratory calibrations. Force-time curves were then constructed. All data were collected and analyzed using custom programs (LabVIEW version 15, National Instruments, Austin, TX, USA). To reduce noise in the signal, a digital low-pass Butterworth filter with a cutoff frequency of 10 Hz was used. From the force-time data, RSI_mod_ was then calculated using procedures outlined by previous authors [8,9,19].

Equation (1)

(1) $$\;{\mathrm{RSI}}_{\mathrm{mod}}=\frac{\mathrm{CMJ}\;\mathrm{height}\;\left(m\right)}{\mathrm{time}\;\mathrm{to}\;\mathrm{takeoff}\;\left(s\right)}\;$$

The CMJ height (JH) was calculated from the vertical displacement of the jumper’s center of mass estimated from flight time [20] and TTT was defined as the time interval between the initiation of the countermovement and the instant when the jumper left the force platform. A threshold of 10 N was used to identify the beginning and end of this period.

### 2.4. Statistical Analyses

Relative and absolute reliability of RSI_mod_ and its constituent variables were assessed using a two-way mixed-effect model intraclass correlation coefficient (ICC) and typical error expressed as a coefficient of variation (CV%) performed between the two CMJ trials. Reliability was found to be acceptable with high test-retest correlation and low CV% for all variables for both males (RSI_mod_: ICC = 0.963, CV% 7.6%; JH: ICC = 0.978, CV% = 4.7%; TTT: ICC 0.899, CV% = 5.4%) and females (RSI_mod_: ICC = 0.967, CV% 7.9%, JH: ICC = 0.978, CV% = 4.1%; TTT: ICC 0.892, CV% = 6.0%), therefore the mean of the CMJ trials was used for all analyses [21,22].

**Figure 1:**
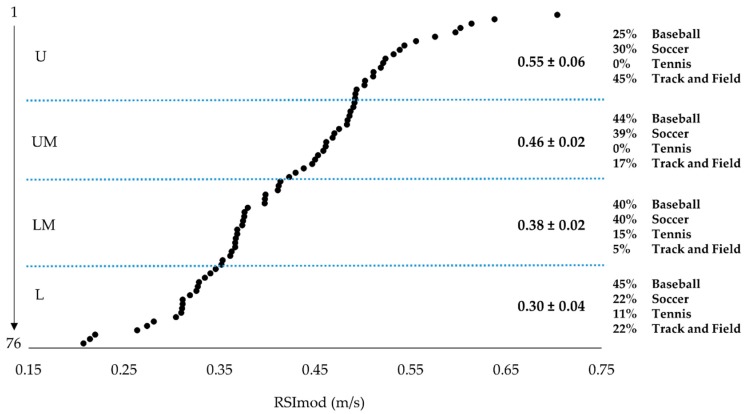
Ranked reactive strength index-modified (RSI_mod_) scores and performance groups for male athletes. Note: U = upper performance group; UM = upper-middle performance group; LM = lower-middle performance group; L = lower performance group.

**Figure 2:**
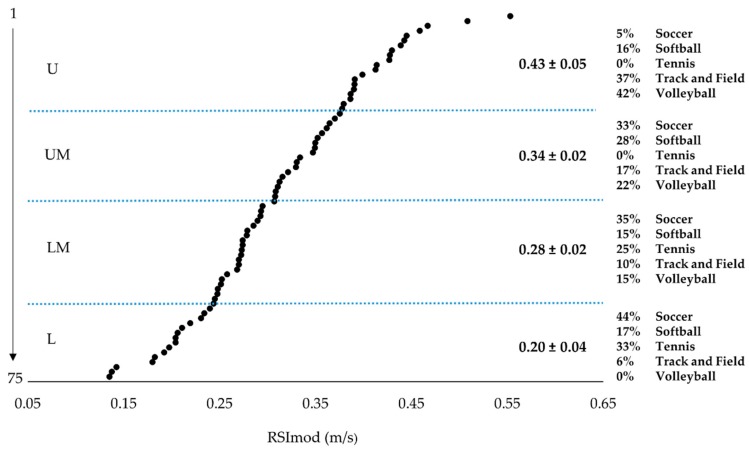
Ranked reactive strength index-modified scores and performance groups for female athletes. Note: U = upper performance group; UM = upper-middle performance group; LM = lower-middle performance group; L = lower performance group.

Once averaged, RSI_mod_ data were aggregated by sex to form male (n = 76) and female (n = 75) groups. RSI_mod_ scores for both males and females were assessed and found to be normally distributed (males: Kolmogorov-Smirnov (D (76) = 0.090, *p* > 0.200; Skewness: 0.170, SE = 0.276; Kurtosis: −0.154, SE = 0.545; females: Kolmogorov-Smirnov (D (75) = 0.064, *p* > 0.200; Skewness: 0.221, SE = 0.277; Kurtosis: −0.272, SE = 0.548). Additionally, no outliers were identified [23,24] for either group. Male and female scales were then constructed using percentile rank. In order to allow for further analysis of the scales, male and female participants were then ranked based on RSI_mod_ scores and then divided into four performance groups using quartiles as cut points (Figure 1 and Figure 2). Independent samples t-tests were used to compare differences in RSI_mod_, JH, and TTT between males and females. To compare differences between each performance group, a series of one-way analysis of variances (ANOVAs) were used to examine the four performance groups with Bonferroni post hoc analysis used when appropriate. Levene’s test was used to assess equality of variance for all group comparisons. To provide an indication of the practical significance of any observed differences, Cohen’s *d* effect sizes were calculated and interpreted in accordance with the scale developed by Hopkins [25]. The critical alpha was set at *p* < 0.05 for all analyses. All statistical analyses were performed using SPSS version 23 (IBM, Armonk, NY, USA) and Microsoft Excel 2013 (Microsoft Corporation, Redmond, WA, USA).

## 3. Results

Descriptive statistics for RSI_mod_ and its constituents for male and female athletes are displayed in Table 1. The preliminary reference value scales for RSI_mod_ for both male and female athletes are displayed in Table 2 and Table 3. Descriptive statistics for male and female athletes stratified by performance group are displayed in Table 4.

Statistically significant differences were observed when comparing male and female athletes with regard to RSI_mod_ (t (149) = 7.09, *p* < 0.001, *d* = 1.15) and JH (t (149) = 8.64, *p* < 0.001, *d* = 1.41). No statistically significant difference was observed for TTT between male and female athletes (t (149) = 0.11, *p* = 0.909, *d* = 0.02). Statistically significant differences were observed for JH and TTT between individual performance groups for both male (JH: (F (_3,72_) = 43.68, *p* < 0.001; TTT: (F (_3,72_) = 12.17, *p* < 0.001) and female athletes (JH: (F (_3,71_) = 63.90, *p* < 0.001; TTT: (F (_3,71_) = 10.11, *p* < 0.001). Specifically, post hoc analyses for the males revealed that JH was statistically different (*p* < 0.05) between all four performance groups with moderate to very large effect sizes observed ranging from *d* = 0.86 to 2.96. For TTT all comparisons were found to be statistically significant with effect sizes ranging from moderate to large (*d* = 0.81 to 1.44), with the exception of the comparisons of upper and upper-middle groups and upper-middle and lower-middle groups that were found not to be statistically different exhibiting moderate (*d* = 0.71) and small (*d* = 0.25) effect sizes, respectively. Similarly, for females, statistically significant (*p* < 0.05) differences were identified between all four performance groups for JH with large to very large effect sizes observed ranging from *d* = 1.26 to 4.50. For TTT, all groups were found to be statistically different (*p* < 0.05) with moderate to large effect sizes observed ranging from *d* = 1.05 to 1.93 with the exception of comparisons of upper and upper middle (*d* = 0.57), upper-middle and lower-middle (*d* = 0.31), and lower-middle and lower (*d* = 0.87) groups, who were determined not to be statistically different, exhibiting small to moderate effect sizes.

## 4. Discussion

The purpose of this analysis was to construct a preliminary scale of reference values for evaluating RSI_mod_. Additionally, this report included an analysis of the variables used to calculate RSI_mod_ in effort to improve the interpretation of this variable. RSI_mod_ values ranged from 0.208 to 0.704 and 0.135 to 0.553 in males and females, respectively. When comparing male and female athletes overall, males exhibited statistically greater RSI_mod_ values. This finding is in agreement with previous research that examined RSI_mod_ differences between male and female athletes [9]. On average, male athletes were found to have RSI_mod_ values 29.8% greater than their female counterparts. Interestingly, when comparing TTT values males and females were found to be quite similar, exhibiting only a 0.2% difference on average. This similarity in TTT between males and females is in agreement with previous investigations examining the temporal structure of the CMJ [26,27]. The observed similarities in TTT values and statistically different RSI_mod_ values, indicates that the primary factor influencing the sex differences observed in RSI_mod_ may be attributed to jump height. In fact, in the present analysis, there was an approximate 30% difference in JH between males and females. The strong influence of JH on RSI_mod_ over TTT can be further illustrated by examining the relationships between these variables. The relationship between RSI_mod_ and TTT was *r* = −0.60 for females and *r* = −0.40 for males, whereas the relationship between RSI_mod_ and JH was *r* = 0.87 and *r* = 0.89 for males and females, respectively. Although not explicitly examined in the present study, there are several reasons why differences in JH may have existed between males and females. Two reasons may be due to differences in muscular strength or in jump strategy. Previous research has indicated that RSI_mod_ displayed strong relationships with maximal isometric strength [28]; however, it should be noted that additional research has indicated that RSI_mod_ differences existed between males and females despite controlling for strength level and using it as a covariate [29]. Furthermore, McMahon et al. [30], as well as Sole et al. [26], have indicated that male participants applied a larger concentric impulse and achieved a greater velocity throughout the concentric phase, which ultimately leads to a greater jump height. It is clear that although differences in RSI_mod_ and JH may exist between males and females, further research is needed to determine the factors that produce these differences.

A secondary analysis of the present data scale involved a comparison of four performance groups representing the upper, upper-middle, lower-middle, and lower male and female athletes, as determined by RSI_mod_. When examining both JH and TTT between the performance groups, only JH was found to be statistically different between all groups. In contrast, no statistical differences and only small to moderate effect sizes were observed when comparing the top two (upper and upper-middle) and middle two (upper-middle and lower-middle) groups for males in TTT values. Similarly for females, no statistical differences and only small to moderate effect sizes were observed between the top two (upper and upper-middle), bottom two (lower and lower-middle), and middle two (upper- and lower-middle) groups. These results indicate that TTT alone was not enough to differentiate between groups, as was the case with JH. As mentioned above, the current findings may be partially explained by muscular strength differences between each group. An abundance of research supports the idea that stronger individuals produce superior jump performances compared to weaker individuals [31]. Another possible explanation for the current findings may be due to differences in musculotendinous stiffness characteristics. Kipp et al. [32] indicated that vertical stiffness was strongly correlated to the traditional measure of reactive strength index (drop jump height/ground contact time). Given that the RSI_mod_ and reactive strength index variants are strongly correlated [33], it is possible that vertical stiffness may also play a role in JH performance during a CMJ. To the authors’ knowledge, no study has yet to investigate the influence of stiffness on RSI_mod_.

The reference values presented in the current study were constructed using athletes from various sporting disciplines. While the use of multiple sports within a single scale may be viewed as a limitation, it should be noted that no other study has used a sample size as large as the one used within the current study. As displayed in Figure 1 and Figure 2, the ranking and grouping of athletes by RSI_mod_ resulted in a disproportionate sport representation within groups. Although this lack of homogeneity within performance groups may be viewed as problematic, it also may simply highlight sport specific differences in jumping strategies as noted by previous reports [34]. Moreover, it should be noted that previous research indicated that within-team differences based on player position may exist when examining RSI_mod_ [35]. Thus, while the RSI_mod_ reference values produced in this study may serve as an initial step in RSI_mod_ comparisons between athletes, it is important for future research to continue to collect normative RSI_mod_ data for different sports and levels of sports so that additional scales may be developed. Furthermore, additional data will allow for the comparison of athletes within a single sport or between different levels of the same sport [1].

Although the scales and analyses provide insight for evaluating RSI_mod_ and its constituent parts, practitioners should be cognizant of some potential limitations of this data. The present scales and analyses used athlete data collected from a single university, and although all athletes were competitive at the NCAA Division I level, this sample may not be representative of all collegiate athletes. Thus, as noted above, it is important that if practitioners see value in using RSI_mod_ as a monitoring tool, additional data for male and female athletes is needed. A second limitation of the present study may be related to the specific data analysis procedures used to calculate RSI_mod_. Although all data were collected using force plates sampling at an optimal sampling frequency [36] and analyzed using identical procedures, the present study used arbitrary thresholds to identify the initiation of the countermovement (unweighting phase) as well as takeoff and landing events. Furthermore, the jump height values used to calculate RSI_mod_ were estimated using time in air. Recently researchers have identified this as potentially problematic as it relates to RSI_mod_ as any errors in identifying TTT or JH will ultimately influence RSI_mod_ [12]. Future analyses should consider using more robust methods [22,37] for identifying these key time points during the jump, in effort to reduce potential error. Although this limitation is valid, within this analysis it may be partially obviated by the fact that all CMJ analyses were completed using identical procedures. 

## 5. Conclusions

The results of the present analysis provide practitioners with preliminary scales of reference values for interpreting RSI_mod_ scores in collegiate athletes. A primary finding of this report was that the difference in RSI_mod_ between male and female Division I athletes is largely attributed to differences in jump height. Interestingly, when compared as a whole, there were no discernable differences in TTT between male and female athletes. Analysis of the RSI_mod_ scales indicate that there are clear performance differences between upper performers and lower male and female performers, in both RSI_mod_ and its constituent parts. Given the importance of comparing performances between individuals, these reference values may provide a valuable resource for practitioners seeking to evaluate their athlete’s performance.

## Figures and Tables

**Table 1:** Descriptive statistics for reactive strength index-modified for male and female athletes (n = 151, mean ± standard deviation.

	Male (n = 76)	Female (n = 75)
RSI_mod_ (m/s)	0.424 ± 0.102	0.314 ± 0.089
JH (m)	0.36 ± 0.07	0.27 ± 0.06
TTT (s)	0.868 ± 0.105	0.870 ± 0.114

Note: RSI_mod_ = reactive strength index-modified; JH = jump height; TTT = time to takeoff

**Table 2:** Reference value scale for reactive strength index-modified for male collegiate athletes (n = 76).

Percentile	RSI_mod_ (m/s)
97	0.630
95	0.604
90	0.547
85	0.523
80	0.508
75	0.492
70	0.487
65	0.476
60	0.461
55	0.448
50	0.419
45	0.398
40	0.376
35	0.369
30	0.366
25	0.352
20	0.331
15	0.316
10	0.308
5	0.257
3	0.216

**Table 3:** Reference value scale for reactive strength index-modified for female collegiate athletes (n = 75).

Percentile	RSI_mod_ (m/s)
97	0.497
95	0.461
90	0.434
85	0.413
80	0.391
75	0.379
70	0.366
65	0.351
60	0.333
55	0.315
50	0.308
45	0.293
40	0.279
35	0.273
30	0.266
25	0.248
20	0.241
15	0.214
10	0.202
5	0.173
3	0.139

**Table 4:** Descriptive statistics for reactive strength index-modified between performance groups (mean ± standard deviation).

Male Performance Group	n	RSI_mod_ (m/s)	JH (m)	TTT (s)	Female Perfomance Group	n	RSI_mod_ (m/s)	JH (m)	TTT (s)
U	20	0.549 ± 0.057	0.43 ± 0.05	0.795 ± 0.087	U	19	0.429 ± 0.045	0.34 ± 0.03	0.792 ± 0.077
UM	18	0.464 ± 0.021	0.39 ± 0.04	0.850 ± 0.067	UM	18	0.343 ± 0.023	0.29 ± 0.04	0.848 ± 0.117
LM	20	0.379 ± 0.019	0.33 ± 0.04	0.871 ± 0.100	LM	20	0.277 ± 0.017	0.24 ± 0.03	0.880 ± 0.092
L	18	0.296 ± 0.044	0.28 ± 0.05	0.964 ± 0.090	L	18	0.203 ± 0.036	0.19 ± 0.03	0.964 ± 0.100

Note: RSI_mod_ = reactive strength index-modified; JH = jump height; TTT = time to takeoff, U = upper performance group; UM = upper-middle performance group; LM = lower-middle performance group, L = lower performance group.
